# SAHA Enhances Synaptic Function and Plasticity *In Vitro* but Has Limited Brain Availability *In Vivo* and Does Not Impact Cognition

**DOI:** 10.1371/journal.pone.0069964

**Published:** 2013-07-26

**Authors:** Jesse E. Hanson, Hank La, Emile Plise, Yung-Hsiang Chen, Xiao Ding, Taleen Hanania, Emily V. Sabath, Vadim Alexandrov, Dani Brunner, Emer Leahy, Pascal Steiner, Lichuan Liu, Kimberly Scearce-Levie, Qiang Zhou

**Affiliations:** 1 Department of Neuroscience, Genentech, Inc. South San Francisco, California, United States of America; 2 Department of Drug Metabolism and Pharmacokinetics, Genentech, Inc., South San Francisco, California, United States of America; 3 PsychoGenics Inc., Tarrytown, New York, United States of America; 4 Department of Psychiatry, Columbia University, New York, New York, United States of America; Georgia Regents University, United States of America

## Abstract

Suberoylanilide hydroxamic acid (SAHA) is an inhibitor of histone deacetylases (HDACs) used for the treatment of cutaneous T cell lymphoma (CTCL) and under consideration for other indications. *In vivo* studies suggest reducing HDAC function can enhance synaptic function and memory, raising the possibility that SAHA treatment could have neurological benefits. We first examined the impacts of SAHA on synaptic function *in vitro* using rat organotypic hippocampal brain slices. Following several days of SAHA treatment, basal excitatory but not inhibitory synaptic function was enhanced. Presynaptic release probability and intrinsic neuronal excitability were unaffected suggesting SAHA treatment selectively enhanced postsynaptic excitatory function. In addition, long-term potentiation (LTP) of excitatory synapses was augmented, while long-term depression (LTD) was impaired in SAHA treated slices. Despite the *in vitro* synaptic enhancements, *in vivo* SAHA treatment did not rescue memory deficits in the Tg2576 mouse model of Alzheimer’s disease (AD). Along with the lack of behavioral impact, pharmacokinetic analysis indicated poor brain availability of SAHA. Broader assessment of *in vivo* SAHA treatment using high-content phenotypic characterization of C57Bl6 mice failed to demonstrate significant behavioral effects of up to 150 mg/kg SAHA following either acute or chronic injections. Potentially explaining the low brain exposure and lack of behavioral impacts, SAHA was found to be a substrate of the blood brain barrier (BBB) efflux transporters Pgp and Bcrp1. Thus while our *in vitro* data show that HDAC inhibition can enhance excitatory synaptic strength and potentiation, our *in vivo* data suggests limited brain availability may contribute to the lack of behavioral impact of SAHA following peripheral delivery. These results do not predict CNS effects of SAHA during clinical use and also emphasize the importance of analyzing brain drug levels when interpreting preclinical behavioral pharmacology.

## Introduction

Histone deacetylases (HDACs) mediate epigenetic changes by decreasing histone acetylation, leading to condensed chromatin structure and decreased transcription [1], [2]. HDACs can also impact cellular functions at various levels through deacetylation of non-histone proteins [3]. SAHA is a HDAC inhibitor that targets Class I and Class IIb Zn^2+^-dependent HDACs, causing increased acetylation. The altered gene regulation induced by SAHA treatment can arrest proliferation of cancer cells [4]. Also known as Vorinostat and marketed as Zolinza, SAHA is currently approved for the treatment of CTCL and is under consideration for treatment of other malignancies [5]–[7]. SAHA is also being considered for non-oncology indications including treatment of malaria infection and depletion of latent HIV reservoirs during antiretroviral therapy [8]–[10]. In the context of brain diseases, recent studies implicate excess HDAC function in Alzheimer’s disease (AD) and suggest decreasing HDAC function with drugs like SAHA could potentially improve cognitive functions [11]. In particular, HDAC2 has been shown to be upregulated in the brains of both AD patients and mouse AD models, and knocking down HDAC2 rescues impaired synaptic plasticity and neurodegeneration-associated memory deficits in an AD mouse model [12]. Furthermore, while transgenic HDAC2 over-expression impairs cognitive functions, HDAC2 knockout mice exhibit enhanced synaptic plasticity and memory function [13]. In another example, reducing HDAC6 function has been shown to protect against neurodegeneration induced by oxidative stress and promote axon outgrowth [14]. As SAHA can block numerous HDACs including HDAC2 and HDAC6, these observations raise the possibility that patients taking SAHA could experience neurological impacts. Such impacts could potentially be beneficial to improve brain function in AD patients. To address this possibility, we explored the impacts of SAHA treatment on neuronal function *in vitro* and on fear memory in AD model mice and general behavioral activity in wild type mice using the SmartCube® System [15]–[17]. While SAHA enhanced synaptic transmission and potentiation *in vitro*, we could not detect any effect of *in vivo* treatment on the behaviors measured. Consistent with a lack of neurobehavioral activity, SAHA exhibited poor brain penetration and was found to be a substrate of brain efflux transporters.

## Materials and Methods

### Ethics Statement

All animal experiments were conducted in accordance with the National Institute of Health Guide for the Care and Use of Laboratory Animals. Experiments performed at Genentech were approved by the Genentech Institutional Animal Care and Use Committee. Experiments performed at PsychoGenics were approved by the PsychoGenics Institutional Animal Care and Use Committee. Experiments performed at Cerebricon were approved by the National Animal Experiment Board of Finland, State Provincial Office of Southern Finland.

### Slice Cultures

Interface cultures of hippocampal slices were made from 7–8 day old Sprague Dawley rats as previously described [18]. Briefly, hippocampi were dissected in minimum essential medium (MEM; Invitrogen, Eugene, OR) with 15 mm HEPES and 10 mm Tris buffer (Invitrogen). Four-hundred micrometer slices were cultured on Millicell CM culture plate inserts (Millipore, Temecula, CA). The culture medium consisted of 50% MEM, 25% HBSS, and 25% horse serum, with 12.5 mM HEPES buffer and penicillin (100 U/ml)/streptomycin (100 µg/ml) (all from Invitrogen). Cultures were maintained in 5% CO_2_, at 37°C. Slices were maintained *in vitro* for one week prior to transfection.

### Electrophysiology

Patch clamp recordings were made in oxygenated Artificial Cerebrospinal Fluid (ACSF) containing (in mM) 127 NaCl, 2.5 KCl, 1.3 MgSO4, 2.5 CaCl2, 1.25 Na2HPO4, 25 NaHCO3, 25 glucose. For voltage-clamp recordings patch pipette internal solution consisted of 120 Cs-methanesulfonate, 20 CsCl, 0.5 EGTA, 10 HEPES, 2.5 MgCl2, 4 Na2ATP, 0.3 NA3GTP, 10 Phosphocreatine, and 5 mM QX-314 Br. Miniature excitatory postsynaptic currents (mEPSCs) were recorded in the presence of 100 µM Picrotoxin (PTX) and 1 µM tetrodotoxin (TTX) at a holding potential of −70 mV. Miniature inhibitory postsynaptic currents (mIPSCs) were recorded in the presence of 10 µM 2,3-Dioxo-6-nitro-1,2,3,4-tetrahydrobenzo[f]quinoxaline-7-sulfonamide (NBQX), 50 µM D-(−)-2-Amino-5-phosphonopentanoic acid (D-AP5) and 1 µM TTX at a holding potential of 0 mV. Significance of differences between mean frequencies or amplitudes of mEPSCs or mIPSCs were assessed using a student’s t-test. Cumulative amplitude distributions were plotted at 10 percentile intervals using values interpolated from 1 pA binned data for each cell. For experiments measuring open NMDAR block synaptic inputs were stimulated locally using a bipolar stimulation electrode and NMDAR EPSCs were isolated using NBQX and PTX and measured at −70 mV with external MgCl2 lowered to 0.5 mM. 20 µM (5S,10R)-(+)-5-Methyl-10,11-dihydro-5H-dibenzo[a,d]cyclohepten-5,10-imine (MK-801) was then added after a stable baseline was achieved and synaptic stimulation was resumed. For synaptic plasticity experiments synaptic inputs were stimulated locally using a bipolar stimulation electrode and EPSCs were measured at −70 mV. To isolate glutamatergic synaptic events and suppress polysynaptic network activity PTX and 10 µM 2-chloroadenosine were added to the ACSF. After a brief baseline (about 5 min), a subthreshold LTP induction protocol was delivered consisting of 100 stimuli at 2 Hz with postsynaptic neurons voltage-clamped at 0 mV. This protocol was selected because we have previously found that no LTP is induced in organotypic slices under basal conditions using this protocol, but robust LTP can be induced when HDAC2 is genetically reduced [19]. LTD was induced using 600 stimuli at 2 Hz with postsynaptic neurons voltage-clamped at −40 mV. Significance of differences in normalized EPSC amplitudes between treatment groups following plasticity induction were assessed using a student’s t-test. For current-clamp recordings internal solution consisted of 120 K-gluconate, 20 KCl, 0.5 EGTA, 10 HEPES, 2.5 MgCl2, 4 Na2ATP, 0.3 NA3GTP and 10 Phosphocreatine. Action potential threshold was measured during the smallest depolarizing current injection steps that triggered spiking. Input resistance was calculated from the steady-state response to the smallest hyperpolarizing current injection steps to avoid contamination from hyperpolarization-induced inward currents (h-current). The h-current “sag” was measured based on the difference between the maximal and steady-state voltage responses to strong hyperpolarizing current injection steps. Significance of differences in all measures between treatment groups were assessed using a student’s t-test.

### Tg2576 Fear Conditioning

Contextual fear conditioning was performed at Cerebricon, Ltd., (Finland). Tg2576 mice overexpressing human amyloid precursor protein (APP) with the ‘Swedish’ mutation, K670N/M671L, under control of the prion promoter, were used. Female Tg2576 mice were treated once a day for 35 days, starting at approximately 5 months of age, with i.p. injections of 25 mg/kg SAHA, 50 mg/kg SAHA or vehicle (DMSO). Injection volume was 10 ml/kg and testing was done a minimum of 2 hours after dosing. Open field testing was performed on dosing day 32. Mice were brought to the experimental room for at least 1 h acclimation prior to testing in the open field. At least two hours after the last dosing, mice were placed in open field chambers equipped with infrared photobeams (Med Associates, Inc., St. Albans VT; 27×27×20.3 cm) and their locomotor activity was monitored for 30 minutes. Contextual fear conditioning was performed on dosing days 33–34 using modification of published protocols [20]. The training and testing were conducted on two consecutive days, using a Coulbourn FreezeFrame system (Coulbourn, Whitehall PA, USA). Day 33 training consisted of placing a mouse in a chamber, bright house light on, and allowing exploration for 2 min. Afterward an auditory cue (1700 Hz, 80 dB, the conditioned stimulus) was presented for 15 s. A 2 s foot shock (1.5 mA, the unconditioned stimulus) was administered for the final 2 s of the auditory cue. This procedure was repeated, and the mouse was removed from the chamber 30 s later. Freezing behavior was recorded during (2 s) and after the shocks (5 s) by a computerized camera tracking system. The next day, the mouse was returned to the same chamber in which the training occurred and freezing behavior was recorded (memory for context). At the end of the 5 min context test, the mouse was returned to its home cage. One hour later, freezing was recorded in a novel environment (altered context) and in response to the cue (memory for cue). Freezing scores for each subject were expressed as a percentage for each portion of the test (memory for context, altered context, memory for cue). Reductions in freezing in Tg2576 mice compared to non-transgenic mice were assessed using a student’s t-test. Effects of treatment (vehicle, 25 mg/kg or 50 mg/kg SAHA) on freezing or open field activity in Tg2576 mice were assessed using a one way ANOVA.

### Bioanalysis of SAHA

Liquid chromatographic-tandem mass spectrometry (LC-MS/MS) was used for the analysis of SAHA. Each brain was homogenized in water (0.25 g/ml) using a OmniPrep homogenizer (OmniPrep, Las Vegas, NV, USA) and each CSF sample was collected into mouse plasma (CSF to plasma 1∶1, v/v). 25 µL of plasma, CSF or tissue sample was extracted using acetonitrile and supernatant was diluted with water and then analyzed using reversed-phase chromatography with an XB-C18, 50×2.1 mm, 5 µm analytical column (Phenomenex). Peak retention time was 1.0 min using the following gradient (time, %B) at flow rate 0.80 mL/min: (0.00 min, 10%) (0.30 min, 10%) (0.60 min, 90%) (1.10 min, 90%) (1.12 min, 10%) (1.60 min, 10%). Mobile phases were water with 0.1% formic acid and acetonitrile with 0.1% formic acid. Mass analysis was conducted on a TSQ Vantage mass spectrometer (Thermo Scientific, West Palm Beach, FL, USA) with multiple reaction monitoring (MRM) mode. SAHA was ionized using an atmospheric pressure chemical ionization (APCI) source operating in the positive ionization mode. Declustering potential and collision energy were 65 V and 12 V, respectively. The MRM transition was m/z 265.3 to m/z 232.2 and the calibration curve range was between 3.05–20,000 ng/mL for plasma and brain, and 0.66–2500 ng/mL for CSF. Calibration curves were fit to a linear regression with 1/×2 weighting for all plasma, brain homogenate and CSF curves.

### Enzymatic Assays

A panel of HDAC enzymatic assays (HDAC1 thru HDAC10) were performed by Nanosyn (Santa Clara, CA) using a Caliper microfluidics platform with fluorescently labeled peptide substrates. SAHA was serially diluted from a top concentration of 10 µM in DMSO and tested at 12 concentrations with 3× dilution intervals. Final DMSO concentration was 1%. Assays used 0.5–5 nM enzyme and 1 µM peptide substrates.

### SmartCube® System

SmartCube experiments were conducted at Psychogenics, Inc (Tarrytown, NY). 10 week old C57BL/6 mice from Taconic (n = 12/group) were either given a single injection or 14 daily injections of SAHA or valproate prior to behavioral phenotyping. On the day of testing, mice were injected with SAHA or valproate 15 minutes prior to testing with the SmartCube® system [15]–[17]. The SmartCube® system can measure numerous spontaneous behaviors and responses to challenges in the same testing environment. The hardware includes force sensors and a number of aversive stimuli used to elicit behavior. Three high-resolution video cameras provide constant 3D view of the mouse in the SmartCube® apparatus throughout the testing period. Digital videos of the subjects were processed with computer vision algorithms to extract over 2,000 independent measures including frequency and duration of behavioral states such as grooming, rearing, etc., and many other features obtained during the test session. These data were compared with a database of therapeutic class signatures. The database comprises 14 classes of drugs with some of the major classes, such as the antidepressant class, comprising several subclasses with representatives of most of the drugs in the market.

### *In vitro* Transporter Assays

Madin-Darby Canine Kidney (MDCK) cells stably transfected with human MDR1 (Pgp) were obtained from the National Institutes of Health, (Bethesda, MD) MDCKII cells transfected with mouse Bcrp1 were obtained from Alfred Schinkel’s lab (Netherlands Cancer Institute, Amsterdam, Netherlands). Both cell lines were maintained in Dulbecco’s Modified Eagle Medium supplemented with 10% FBS and 5 µg/mL Plasmocin and were harvested with trypsin and seeded on Millipore Millicell-24 well plates at initial concentrations of 1.30×10^5^ cells/mL and 2.50×10^5^ cells/mL, respectively. Cell monolayers were equilibrated in transport buffer (HBSS with 10 mM Hepes, pH 7.4) for 30 minutes at 37°C with 5% CO2 and 95% relative humidity, prior to the experiment. Dose solutions were prepared in transport buffer and consisted of SAHA (5 µM) and the monolayer integrity marker lucifer yellow (100 µM) in the presence and absence of the MDR1 inhibitor Elacridar (2 µM) or the Bcrp1 inhibitor Ko-143 (1 µM). The dose solutions were added to the donor chambers and transport buffer (with and without inhibitor) was added to receiver chambers. The transport of SAHA was examined in the apical to basolateral (A–B) and basolateral to apical (B-A) directions. The receiver chambers were sampled (50 µL) at 60, 120, and 180 min and were replenished with fresh transport buffer after the 60 and 120 min samplings. Lucifer yellow permeability was used as a marker of monolayer integrity during the experiment. SAHA concentrations in the donor and receiving compartments were determined by LC-MS/MS analysis. The apparent permeability (P_app_) in the apical to basal A–B and basal to apical B-A directions, was calculated as follows: P_app_ = (dQ/dt)•(1/AC_0_), where: dQ/dt = rate of compound appearance in the receiver compartment; A = Surface area of the insert; and C_0_ = Initial substrate concentration at T_0_. The efflux ratio (ER) was calculated as (P_app_ B–A/P_app_ A–B).

## Results

### *In vitro* SAHA Treatment Enhances Excitatory Synaptic Function

**Figure 1:**
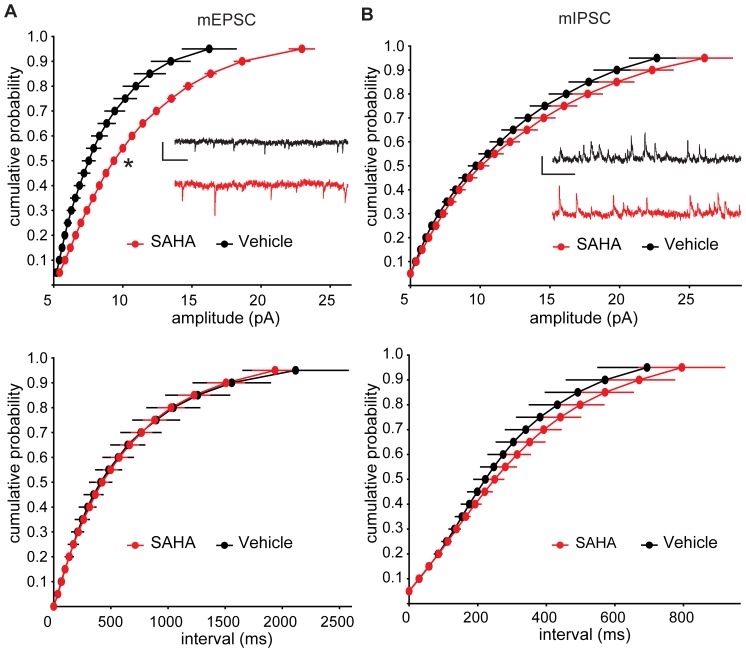
Excitatory synaptic function is selectively enhanced in CA1 of hippocampal brain slices following *in vitro* SAHA treatment. (A) The median amplitude of mEPSCs was significantly increased in SAHA-treated slices (p<0.05, n = 14 vehicle, 14 SAHA), while there was no significant change to mEPSC frequency as measured by the median interval between events (p>0.05). Example mEPSC traces from vehicle and SAHA treated slices are shown inset (scale bar represents 10 pA and 250 ms). (B) SAHA treatment did not significantly alter the amplitude or frequency of mIPSCs (p>0.05, n = 14, 11). Example mIPSC traces from vehicle and SAHA treated slices are shown inset (scale bar represents 10 pA and 500 ms). All data points are plotted as mean ±SEM.

Previous studies have reported that HDAC inhibitors can alter synaptic function when applied to acutely prepared brain slices for as little as 10–90 minutes [21]–[23], a time frame that is unlikely to involve effects of transcriptional alterations due to HDAC inhibition. We wanted to explore the consequences of ongoing exposure to SAHA so as to better reflect therapeutic dosing which is expected to include transcriptional changes that could take hours or days to become evident. Therefore we used organotypic hippocampal brain slices that were treated with 0.5 µM SAHA for 3–4 days prior to assessing synaptic function. First we examined spontaneous miniature excitatory and inhibitory postsynaptic currents (mEPSCs and mIPSCs) in CA1 pyramidal neurons following treatment with SAHA or vehicle (DMSO). These experiments revealed an increase in the amplitude but not frequency of mEPSCs, indicating enhanced excitatory synaptic strength following SAHA treatment (Figure 1A). On the other hand, no changes in the amplitude or frequency of mIPSCs were detected (Figure 1B). These results indicate SAHA treatment enhances excitatory synaptic transmission without affecting inhibitory synaptic transmission. Thus, while SAHA could potentially cause multiple impacts on synaptic function via inhibition of various individual HDAC isozymes, the net impact of several days of pan-HDAC inhibition, including any compensatory responses, is manifested as a selective enhancement of basal excitatory synaptic function.

### Presynaptic Function and Intrinsic Excitability are Unaffected by *in vitro* SAHA Treatment

**Figure 2:**
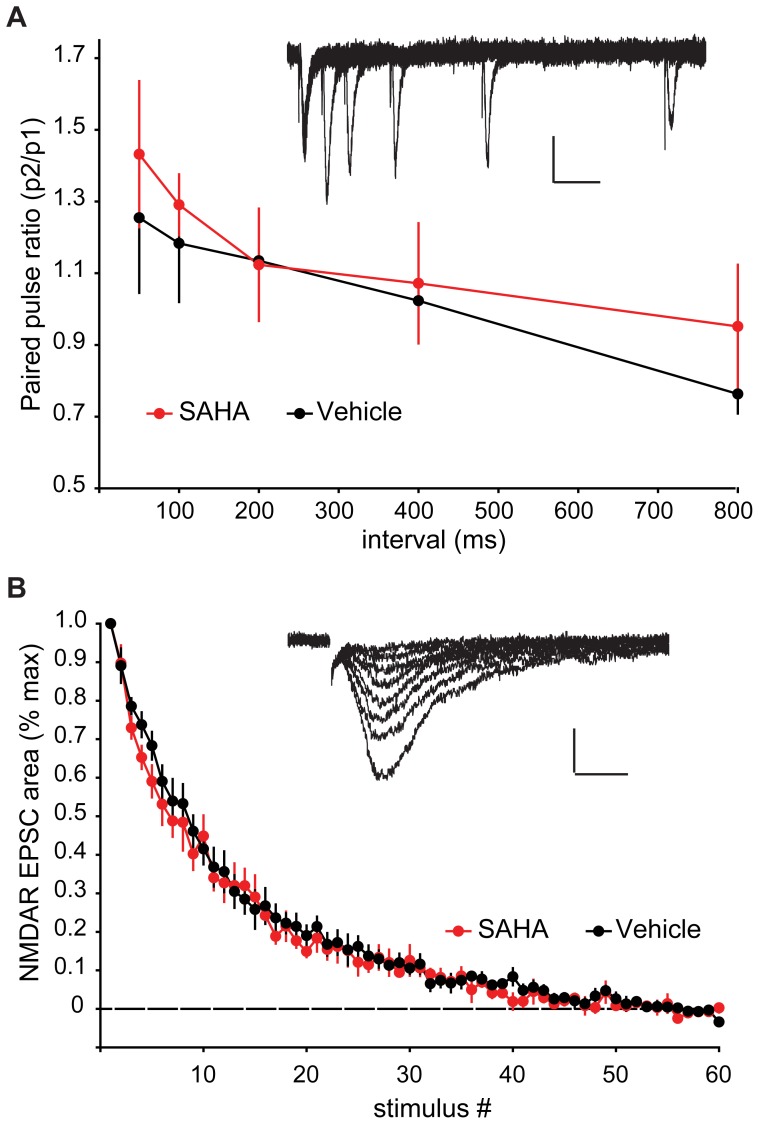
Measures reflecting presynaptic function are normal in SAHA treated slices. (A) The paired-pulse ratio (PPR; amplitude p2/p1) was not significantly different at 50, 100, 200, 400 or 800 ms intervals between stimuli in SAHA vs vehicle treated slices (p>0.05, n = 5 vehicle, 4 SAHA). Example vehicle EPSCs are shown inset (scale bar represents 20 pA and 100 ms). (B) The rate of NMDAR EPSC blockade by MK-801 during repetitive stimulation was not different between SAHA vs vehicle treated slices. (p>0.05, n = 7 vehicle, 7 SAHA). Example vehicle NMDA EPSCs are shown inset (scale bar represents 50 pA and 20 ms). Data are plotted as mean ±SEM.

**Figure 3:**
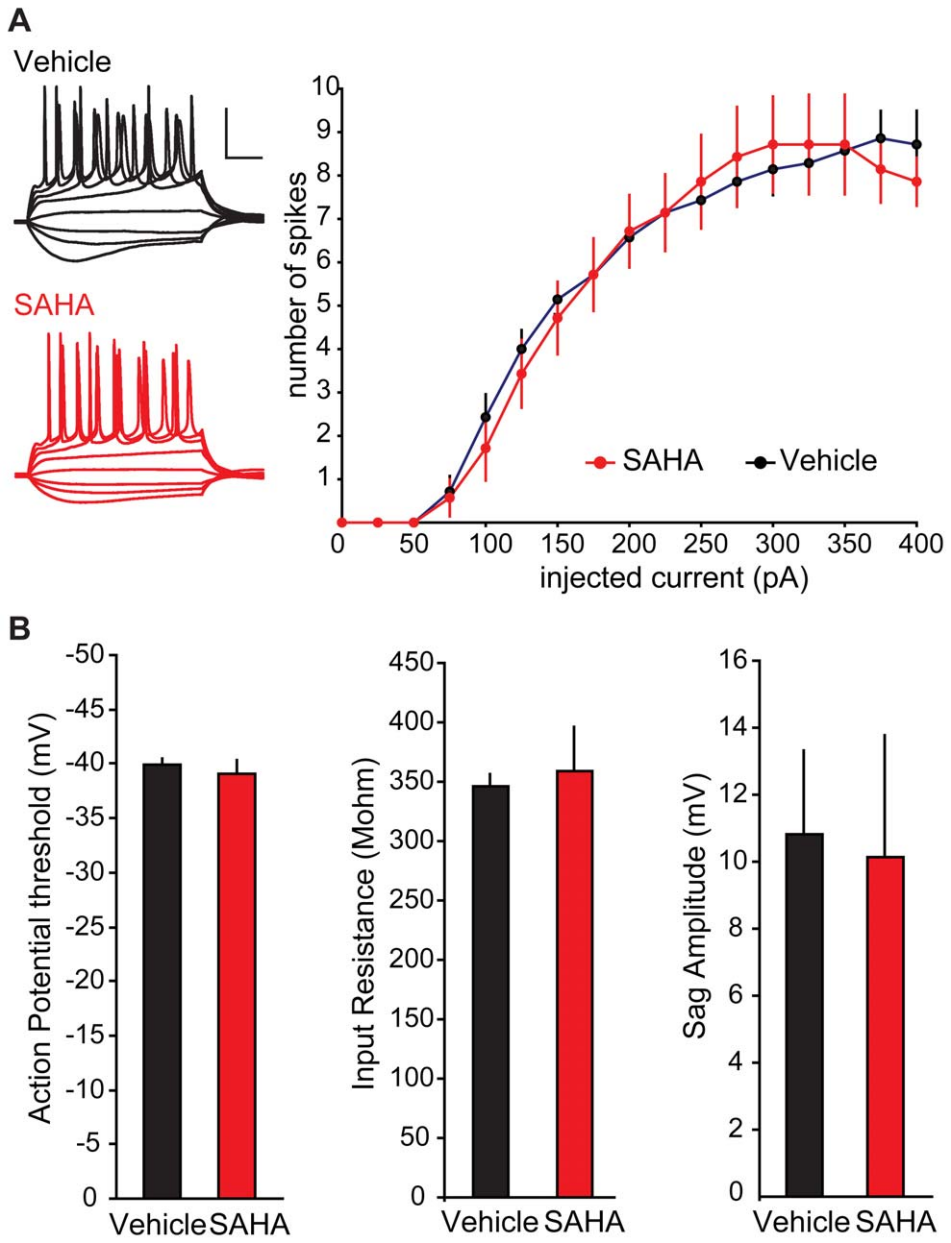
Intrinsic membrane properties are unaltered by SAHA treatment. (A) Representative traces from vehicle (black) and SAHA (red) treated slices during a series of hyperpolarizing and depolarizing current injection steps (scale bar represents 20 mV and 100 ms). There was no difference between vehicle and SAHA treated slices in the number of action potentials elicited by 500 ms current injection pulses at any of the current injection levels (p>0.05, n = 7,7). (B) Action potential threshold, input resistance, and membrane sag reflecting the hyperpolarization-induced inward current, were all unaltered following SAHA treatment (p>0.05). Data are plotted as mean ±SEM.

The increased amplitude but not frequency of mEPSCs is consistent with postsynaptic rather than presynaptic changes following SAHA treatment. To test whether presynaptic functions are altered by SAHA, we measured parameters that are sensitive to alterations in presynaptic release probability. First we examined short-term plasticity, including paired-pulse facilitation at short inter-stimulus intervals, and paired-pulse depression at longer inter-stimulus intervals, measures that are dependent on presynaptic release probability [24]. These recordings showed no differences in paired-pulse facilitation or paired-pulse depression between SAHA and vehicle treated slices (Figure 2A). Second we examined the rate of use-dependent blockade of isolated NMDAR-mediated EPSCs by the NMDAR open channel blocker MK-801. The rate of blockade by MK-801 is dependent on presynaptic release probability and hence serves as another read-out of presynaptic function. The rate of blockade also did not show any difference between SAHA and vehicle treated slices (Figure 2B). Together these results support the conclusion that the enhanced mEPSC amplitudes result from postsynaptic alteration in the absence of any significant changes to presynaptic release probability. To look for non-synaptic alterations following SAHA treatment, we examined the intrinsic excitability of CA1 neurons. No changes were observed in: 1) the number of action potentials evoked by various levels of current injection; 2) action potential threshold; 3) input resistance; or 4) the membrane sag caused by hyperpolarization-activated inward currents (Figure 3). Together these results suggest that SAHA does not alter intrinsic neuronal excitability.

### *In vitro* SAHA Treatment Augments LTP and Blocks LTD

**Figure 4:**
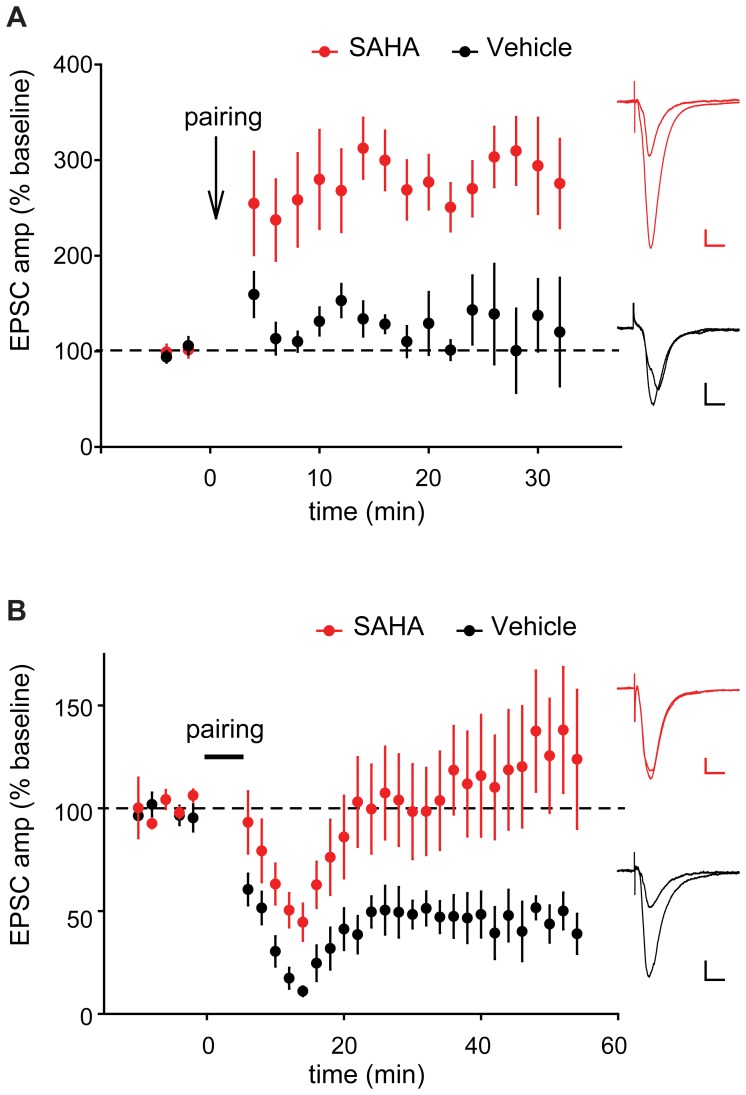
SAHA treated slices exhibit enhanced induction of LTP and impaired LTD. (A) An induction protocol that was subthreshold in vehicle treated slices readily evoked LTP in SAHA treated slices (p<0.05, n = 5,5). Example traces before and after LTP induction are shown in red for SAHA and black for vehicle treated slices (scale bars represent 20 pA and 20 ms). (B) An induction protocol that readily induced LTD in vehicle treated slices could not produce LTD in SAHA treated slices (p<0.05, n = 9 vehicle, 8 SAHA). Example traces before and after LTD induction are shown in red for SAHA and black for vehicle treated slices (scale bars represent 25 pA and 20 ms). Data are plotted as mean ±SEM.

We tested whether treating slice cultures with SAHA for several days could alter synaptic plasticity. Robust LTP was observed in SAHA-treated slices using a subthreshold LTP-induction protocol that did not evoke significant synaptic potentiation in vehicle-treated slices (Figure 4A). This indicates that SAHA treatment can lower the threshold for inducing LTP. On the other hand no LTD was observed in SAHA-treated slices using an induction protocol that caused significant LTD in vehicle-treated slices (Figure 4B), indicating SAHA treatment blocks LTD induction and/or expression. Together these results demonstrate that SAHA treatment alters synaptic plasticity in a manner that enhances potentiation and limits depression of excitatory synapses.

### *In vivo* SAHA Treatment does not Rescue Fear Conditioning Deficits in Tg2576 Mice

**Figure 5:**
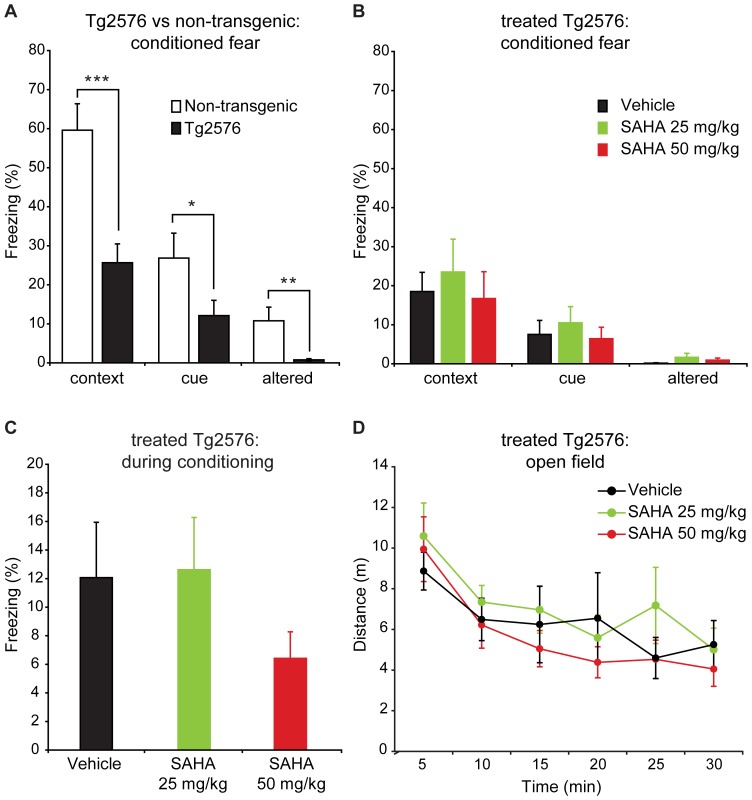
Fear memory deficits in Tg2576 mice are not rescued by SAHA treatment. A) Compared to non-transgenic littermates (n = 15), Tg2576 (n = 14) mice showed significantly less freezing than wt mice when returned to the context in which conditioning occurred (context, p<0.001), when placed in an altered context (altered, p<0.01), or in response to the cue used for conditioning (cue, p<0.05). B) Tg2576 mice were treated daily for 33 days prior to, as well as during fear conditioning with either vehicle (n = 14), 25 mg/kg SAHA (n = 13), or 50 mg/kg SAHA (n = 13). There was no effect of treatment on the percentage of time Tg2576 mice spent freezing in response to the context, altered context, or cue (p>0.05). C) There was no effect of treatment on the percentage of time spent freezing during conditioning (p>0.05). D) There was no effect of treatment on the distance traveled in the open field test (total distance = 38.0±7.7 m for vehicle, 39.6±6.1 m for 25 mg/kg SAHA, and 34.2±5.2 m for 50 mg/kg SAHA, p>0.05). All data are plotted as mean ±SEM.

LTP is considered to be a key substrate of memory formation [25], [26] and the hippocampus plays a critical role in contextual fear memory [27], [28]. Tg2576 AD model mice exhibit impaired contextual fear conditioning that correlates with impaired hippocampal LTP [29]. Therefore the enhanced LTP seen in the hippocampus following *in vitro* treatment with SAHA could potentially translate into improvement of contextual fear conditioning in the Tg2576 mice. Furthermore, previous studies have suggested that HDAC inhibition using various pan-HDAC inhibitors can rescue deficits in contextual fear conditioning in AD models [30]–[33], and SAHA injection in particular has been reported to enhance fear memory [13], [30]. Therefore we tested if treatment with SAHA could rescue the robust deficits in fear memory seen in the Tg2576 mice (Figure 5A). To reflect ongoing therapeutic dosing, mice were given daily i.p. injections of 25 or 50 mg/kg SAHA or vehicle for 35 days and were tested for conditioned fear memory during the final days of dosing. Surprisingly, these experiments did not reveal any improvement of contextual fear memory, with no effect of SAHA treatment on the time spent freezing in response to either the conditioned context or the conditioned cue (Figure 5B). Freezing in response to the unconditioned stimulus (foot shock) was also unaffected (Figure 5C), indicating that SAHA did not alter the response to the shock. Locomotor activity can be a confound in fear conditioning experiments, so we also examined activity in the open field, but again saw no effect of SAHA treatment (Figure 5D). One potential explanation for the lack of behavioral impact of *in vivo* SAHA treatment is poor CNS exposure of SAHA following peripheral delivery. Therefore we analyzed the concentration of SAHA in samples taken from the 50 mg/kg treatment group 1 hour post injection on day 35 of treatment. These measurements revealed that while total plasma levels of SAHA were 1.53±0.80 µM, and CSF levels were 0.61±0.38 µM, levels in the brain were below the lower limit of quantification (LLOQ) of 0.17 µM in these samples. This bioanalysis suggests poor pharmacokinetic properties of SAHA could potentially explain the lack of efficacy.

### SAHA has Limited Brain Availability Following Peripheral Administration

**Figure 6:**
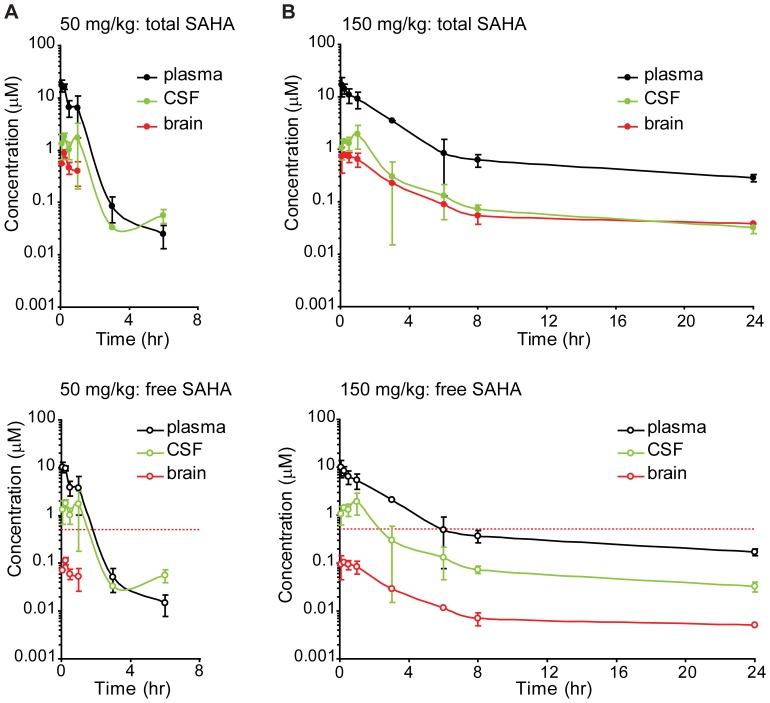
Pharmacokinetic analysis of SAHA following i.p. injection. A) Bioanalysis of the time course of total (top) and unbound (bottom) plasma, CSF, and brain levels of SAHA following a single 50 mg/kg ip injection (n = 3 mice/time point). The dotted red lines represent the SAHA concentration imposed on the *in vitro* slice cultures for the electrophysiological studies. B) Total (top) and unbound (bottom) SAHA levels are shown following a 150 mg/kg ip injection (n = 3/time point). All data is shown as mean ± SD.

To better understand the bioavailability of SAHA we performed a pharmacokinetic study of the time course of both total and unbound SAHA concentrations in the plasma, CSF, and brain following 50 mg/kg i.p. injections (Figure 6A). This analysis revealed: 1) a significant amount of SAHA was bound to protein (40.7% plasma, 87.0% brain); 2) SAHA was rapidly cleared and undetectable in the brain at time points beyond 1 hour (LLOQ = 0.018 µM); and 3) brain levels of SAHA were much lower than plasma levels, with total and free brain-to-plasma ratios of 0.04 and 0.01 respectively (area under the curve up to the last measureable time point; AUC_last_ ratios). To determine if a higher dose could enhance brain exposure, pharmacokinetic analysis was repeated following 150 mg/kg SAHA injections (Figure 6B). While the higher dose did not increase the maximal observed concentration (C_max_) of free SAHA in the brain (0.11±0.02 µM for 50 mg/kg vs 0.10±0.01 µM for 150 mg/kg), the AUC_last_ was increased from 0.07 to 0.36 hr·µM. Overall the pharmacokinetic analysis demonstrates low brain concentrations of free SAHA, and shows that increasing the dose can prolong SAHA exposure but does not enhance the peak concentration achieved in the brain.

### SAHA Inhibits Different HDAC Isozymes with variable Potency

To help interpret the potential impact of the free brain SAHA concentrations achieved by *in vivo* SAHA injection, we examined the potency of SAHA against the different HDAC isozymes as determined by *in vitro* enzymatic assays (Table 1). Based on this data, the free brain SAHA C_max_ of 0.11 µM (which was transiently achieved in the *in vivo* pharmacokinetic studies) exceeded the IC_50_ values of the Class I HDACs, HDAC1 (0.061 µM) and HDAC3 (0.019 µM) but did not reach the IC_50_ values of HDAC2 (0.251 µM) or HDAC8 (0.827 µM). The IC_50_ values of all of the class IIa HDACs were far greater than the *in vivo* Cmax with values all >10 µM. On the other hand the class IIb HDACs had very low IC_50_ values with the Cmax of the *in vivo* studies above the IC_50_ for both HDAC6 (0.009 µM) and HDAC10 (0.029 µM). Moreover the free brain concentrations remained above the HDAC6 IC_50_ for at least 6 hours after the 150 mg/kg dose. Overall these results suggest *in vivo* injections do not result in significant inhibition of the HDAC2 isozyme that is critically involved in fear memory and in deficits in AD model mice [12], [13]. Furthermore, the *in vivo* injections of SAHA could only transiently impact a subset of the other HDAC isozymes with the exception of HDAC6, which could potentially be inhibited for a more prolonged period following higher SAHA doses. In contrast the continuous exposure of brain slices to 0.5 µM SAHA in our *in vitro* studies should be sufficient to broadly inhibit all of the class I HDAC isozymes, including HDAC2, as well the Class IIb isozymes.

**Table 1:** SAHA IC_50_ values for each HDAC isozyme are shown as measured using *in vitro* enzymatic assays.

	Class I				Class IIa				Class IIb	
	HDAC1	HDAC2	HDAC3	HDAC8	HDAC4	HDAC5	HDAC7	HDAC9	HDAC6	HDAC10
IC_50_(µM)	0.061	0.251	0.019	0.827	>10	>10	>10	>10	0.009	0.029
(CI)	(0.004)	(0.016)	(0.001)	(0.078)					(0.001)	(0.002)

Values are in µM and confidence intervals are in parentheses.

### High-content Phenotyping does not Reveal a Significant Neurobehavioral Impact of *in vivo* SAHA Treatment

**Figure 7:**
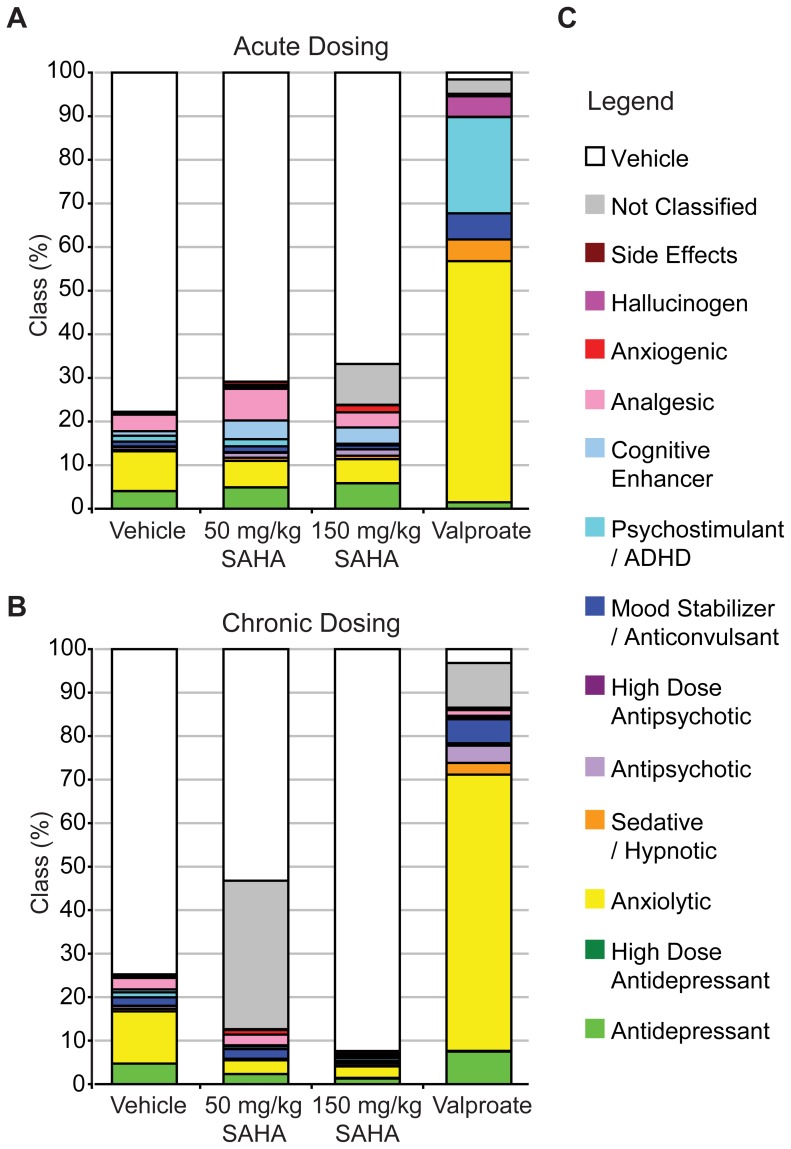
Acute or chronic SAHA treatment does not produce significant drug class activity signatures as assessed by the SmartCube®. A. Groups of mice were treated acutely with a single injection of 50 mg/kg or 150 mg/kg SAHA or vehicle. In addition, a group was treated with valproate (225 mg/kg). Both does of SAHA were behaviorally inactive without a clear therapeutic signal. In contrast valproate was behaviorally active (p<0.001, discrimination index = 100%) with a strong anxiolytic signature and a mild psychostimulant signature. B. Groups of mice were treated daily for 14 days with SAHA or Valproate. While the lower dose of SAHA appeared behaviorally active (p<0.001, discrimination index = 88%), the activity was not consistent with any known therapeutic signal and the higher dose was not behaviorally active. In contrast valproate showed a strong behavioral activity (p<0.001, discrimination index = 98%) with a predominantly anxiolytic signature. C. The legend shows the 15 classes of behavioral activity that were assessed.

That the free brain concentration of SAHA did not even briefly reach the IC_50_ of HDAC2 which is thought to be critical in fear memory and memory impairments in AD model mice [12], [13] is consistent with the lack of improvement of fear memory observed in the Tg2576 mice. Nonetheless, given the expected effects of HDAC inhibition on gene transcription, it is possible that even a transient inhibition of the other class I HDACs or Class IIb HDACs could be sufficient to result in some behavioral impacts, especially at higher doses or after prolonged treatment. Therefore, to broadly asses any behavioral effects of SAHA in C57BL/6 mice we employed high-content behavioral phenotyping using the SmartCube® technology [15]–[17]. This technology assesses behavioral responses to test compounds and determines the class of CNS activity corresponding to any observed behavioral activity. For this study 50 mg/kg or 150 mg/kg doses of SAHA were given to wild type mice prior to SmartCube® testing. A known behaviorally relevant dose of valproate (225 mg/kg) was used as a positive control for the sensitivity of the assay, although it should be noted that this drug has activity on many different targets, and its impacts on behavior should not be assumed to be due to HDAC inhibition. Groups of mice were tested following a single injection or following 14 days of injections to allow for any chronic effects of treatment to develop. There were no significant behavioral alterations observed following single injections of 50 mg/kg or 150 mg/kg SAHA (Figure 7A). Following chronic treatment, the only significant behavioral activity detected for SAHA was in the lower dose group and did not correspond to the signature of any known pharmacological class (Figure 7B). The lack of any behavioral activity in the higher SAHA dose group argues against the unclassified activity in the lower dose group reflecting a CNS effect of chronic SAHA treatment. In contrast, valproate showed strong anxiolytic and mild psychostimulant class behavioral activity after acute treatment (Figure 7A) and also exhibited a strong anxiolytic activity signature in chronically treated mice, confirming sensitivity of this assay (Figure 7B). Overall these results do not support a behavioral effect of up to 150 mg/kg SAHA following acute or chronic i.p. injections.

### SAHA is a Substrate of Brain Efflux Transporters

**Figure 8:**
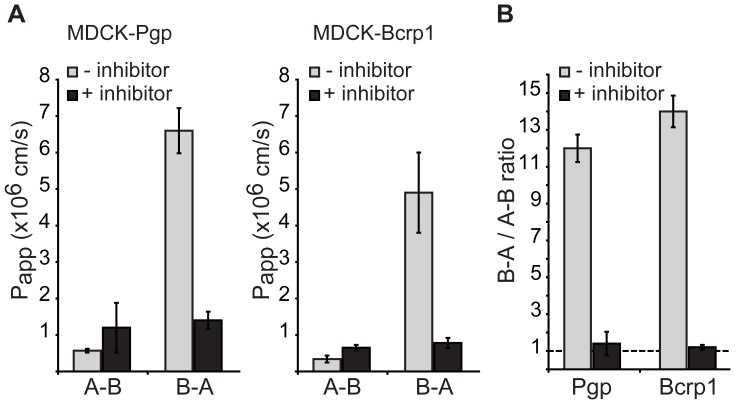
SAHA is a substrate of the brain efflux transporters Pgp and Bcrp1. A) *In vitro* transporter assays using MDCK cell monolayers expressing Pgp or Bcrp1. Apical to basolateral (A–B) and basolateral to apical (B-A) apparent permeability (P_app_) rates are shown either without inhibitors or in the presence of the Pgp inhibitor Elacridar (2 µM) or the Bcrp1 inhibitor, Ko-143 (1 µM). B) The efflux ratios (B–A P_app_/A–B P_app_) are shown with or without the respective inhibitors for Pgp or Bcrp1. All data is shown as mean ± SD.

Given the lack of behavioral impacts of SAHA, we wanted to better understand the causes of limited free SAHA in the brain. One possibility is that low levels of SAHA in the brain could be due to active export of this drug across the BBB. To test if SAHA is a substrate of brain efflux transporters we performed *in vitro* transporter assays using monolayers of MDCK cells expressing human P-glycoprotein (Pgp; Multidrug Resistance Protein 1) or mouse Breast Cancer Resistance Protein (Bcrp1), two important ATP-binding cassette (ABC) gene family members that are present in the BBB and often limit drug exposure in the brain [34], [35] (Figure 8A). These experiments showed efflux ratios for SAHA of 12±0.75 for Pgp and 14±0.86 for Bcrp1 compared to ratios of 1.4±0.64 and 1.2±0.13 in the presence of the respective transporter inhibitors (Figure 8B). These results suggest SAHA is a substrate of both Pgp and Bcrp1 and that active efflux likely makes a major contribution to the low brain distribution of SAHA following peripheral delivery.

## Discussion

Our *in vitro* electrophysiological analysis using cultured brain slices demonstrates that directly treating brain tissue with SAHA can impact both basal synaptic function and synaptic plasticity. The enhanced mEPSC amplitude but not frequency, together with unchanged short-term plasticity and use-dependent NMDAR blockade rate, strongly support a postsynaptic enhancement of excitatory synapses. At the same time we did not detect changes to inhibitory synaptic transmission, which suggests SAHA treatment leads to an overall shift in the balance between excitation and inhibition in the neural network. These results are consistent with studies of HDAC2 KO mice that show increased spine density in CA1 pyramidal neurons, which has been interpreted as reflecting increased excitation and attributed to HDAC2 regulation of genes involved in synapse formation and plasticity [13]. At the same time it is important to keep in mind that the state of synaptic and neuronal function measured after several days of drug treatment reflects the combined effects of inhibiting multiple HDAC isozymes by SAHA, as well as any potential network-wide compensatory processes. For example we have recently reported that selective HDAC2 knockdown in individual neurons in this preparation not only enhances excitation, but also reduces GABAergic synaptic inhibition in a cell-autonomous manner [19]. The lack of a change in synaptic inhibition observed after several days of SAHA treatment suggests that either blockade of isozymes other than HDAC2 enhance inhibition so as to negate HDAC2-mediated reductions, and/or that network-wide compensatory mechanisms counteract any initial cell-autonomous changes in synaptic inhibition. Thus while we cannot discriminate isozyme-specific contributions to the neurophysiologcal alterations observed following SAHA treatment, the net impact of SAHA treatment, is an altered functional state of the network featuring enhanced excitation.

Our *in vitro* brain slice experiments also showed that, similar to observations of enhanced LTP following very acute treatment with SAHA or other pan-HDAC inhibitors [21]–[23], enhanced LTP occurs during more prolonged HDAC inhibition. Furthermore we extended the examination of synaptic plasticity to LTD and found that SAHA treatment impaired this form of synaptic plasticity. Therefore in addition to enhancing basal excitatory synaptic transmission, *in vitro* SAHA treatment also makes it easier to potentiate excitatory synapse and harder to weaken them, thus shifting the balance towards synaptic potentiation/stabilization rather than weakening/elimination. Overall these results predict that HDAC inhibition will promote excitation and network activity, which could be beneficial in cases of cognitive impairment featuring weakened synaptic function, reduced synaptic potentiation, and loss of excitatory synapse, as is the case in mouse models of AD.

Despite expectations from previous studies of contextual fear conditioning using the HDAC inhibitor phenylbutyrate in Tg2576 mice [31], or using various HDAC inhibitors including SAHA in double transgenic AD model mice (over-expressing the same ‘swedish’ mutant APP as the Tg2576 mice as well as mutant presenilin 1) [30], we did not observe a significant rescue of contextual fear conditioning in Tg2576 mice treated with 25 mg/kg or 50 mg/kg SAHA. Bioanalysis of samples taken from these mice 1 hour after dosing on day 35 of daily injections revealed that SAHA levels were low in the CSF and undetectable in the brain. Detailed pharmacokinetic analysis demonstrated high binding, rapid clearance and very low levels of free SAHA in the brain. Consistent with the low levels of free SAHA that were only transiently detectable in the brain, *in vitro* transporter assays showed that SAHA is a substrate for the brain export transporters Pgp and Bcrp1. While previous studies have proposed that brain SAHA exposure could be increased by using a vehicle that enhances solubility [36], our results suggest peripheral delivery of SAHA will be hampered in achieving brain exposure due to active export across the BBB regardless of peripheral solubility.

Based on our *in vitro* enzymatic assay results, peak free brain levels would appear insufficient to significantly impact HDAC2, HDAC8 or any of the Class IIa HDACs. However other reports using *in vitro* assays have sometimes reported higher potency for SAHA (with IC_50_ or Ki values in the 1–200 nM range compared to our IC_50_ of 251 nM for HDAC2, for example) [37]–[39], which emphasizes the dependence of potency measurements on *in vitro* assay conditions, and the uncertainty over the precise potency of *in vivo* SAHA for HDACs in the brain. In addition, based on our *in vitro* assays, the free brain levels did at least transiently exceed the IC_50_ values for HDAC1, HDAC3, HDAC6, and HDAC10. Therefore, taken together with reports of increased histone acetylation following peripheral delivery of SAHA [36], [40], this suggests that despite limited availability of free drug, some degree of target engagement for some HDAC isozymes may occur in the brain. While the roles of HDACs other than HDAC2 in neurobehavior are not well understood, it is possible their inhibition could affect behavior of wildtype mice even if HDAC2 is not significantly inhibited. However, broadly screening for effects of SAHA injections in wildtype mice using the SmartCube® system failed to detect any impact of SAHA: no relevant neurobehavioral activity consistent with any class of drug action was evident following either a single injection or 14 days of dosing with 50 mg/kg or 150 mg/kg SAHA. Overall the lack of efficacy in Tg2576 mice or any behavioral impacts in wt mice is likely contributed to by the very poor pharmacokinetic profile of SAHA in the brain. However, from our data we cannot exclude the possibility that sufficient HDAC inhibition is achieved to result in significant alteration in histone acetylation levels, but there is simply no effect of such altered acetylation on the behavioral assays we conducted.

Our observations of low free SAHA in the mouse brain and no behavioral impact of SAHA following peripheral injection are at odds with previous studies reporting enhanced contextual fear conditioning memory in mice following peripheral SAHA delivery [13], [30]. At the same time it is worth noting that our results do not contradict the broader pharmacological evidence for a role of HDACs in memory, as enhanced contextual fear memory has been demonstrated using various other HDAC inhibitors which likely have distinct pharmacokinetic properties from SAHA [22], [30]–[33], [41], [42]. In addition, studies using direct infusion of SAHA into the brain [21], [43], or genetic reduction of HDAC isozymes [12], [13] have also demonstrated the importance of HDAC function in memory formation. Therefore our *in vitro* results are in agreement with previous studies suggesting HDAC inhibition impacts synaptic function, but our *in vivo* results argue against the usefulness of SAHA in particular in causing such impacts *in vivo*, which is relevant given the clinical use of this compound. Our results also emphasize that the interpretation of behavioral pharmacology data should always include careful analysis of brain drug levels in order to identify true negative results and eliminate false positive results.

It is worth noting that compromised BBBs could allow brain effects of SAHA not seen in our *in vivo* studies. For example SAHA has been reported to be efficacious in models of glioblastoma [44], [45] and is currently being tested in clinical trials for patients with brain tumors. One possibility is that the efficacy of SAHA in preclinical glioma models is facilitated by compromised BBB function in the area of the tumor. Supporting this, tumor cells have been demonstrated to induce changes in the composition of the basal lamina and in astrocytic components of the neurovascular unit and increase vascular permeability in mouse brains [46], [47]. Thus while our results do not predict a neurological impact of SAHA in CTCL patients, who are unlikely to have compromised BBBs, it is plausible that SAHA would be more available in the brain of glioblastoma patients. At the same time, our results suggest that in addition to issues with toxicity during chronic dosing, SAHA is also unlikely to be efficacious in neurological conditions such as AD which generally feature intact BBB function. The finding of limited access of SAHA to the brain also has implications beyond neurological indications. For example, given recent excitement around using SAHA to disrupt HIV latency in order to eradicate infection [8], our results suggest it will be critical to consider the extent of HIV brain reservoirs [48], [49]. Overall our results highlight that while HDAC inhibition can impact neuronal function, efforts to develop HDAC inhibitors for targeting CNS indications need to focus not only on reducing toxicity and achieving selectivity, but also on avoiding compounds that are substrates for efflux transporters in order to allow adequate brain exposure.

## References

[1] Jenuwein T, Allis CD (2001) Translating the histone code. Science 293: 1074–1080. 11498575 10.1126/science.1063127

[2] Strahl BD, Allis CD (2000) The language of covalent histone modifications. Nature 403: 41–45. 10638745 10.1038/47412

[3] Spange S, Wagner T, Heinzel T, Kramer OH (2009) Acetylation of non-histone proteins modulates cellular signalling at multiple levels. Int J Biochem Cell Biol 41: 185–198. 18804549 10.1016/j.biocel.2008.08.027

[4] Sakajiri S, Kumagai T, Kawamata N, Saitoh T, Said JW, et al (2005) Histone deacetylase inhibitors profoundly decrease proliferation of human lymphoid cancer cell lines. Exp Hematol 33: 53–61. 15661398 10.1016/j.exphem.2004.09.008

[5] Duvic M, Vu J (2007) Update on the treatment of cutaneous T-cell lymphoma (CTCL): Focus on vorinostat. Biologics 1: 377–392. 19707308 PMC2721288

[6] Duvic M, Vu J (2007) Vorinostat: a new oral histone deacetylase inhibitor approved for cutaneous T-cell lymphoma. Expert Opin Investig Drugs 16: 1111–1120. 10.1517/13543784.16.7.1111 17594194

[7] Martinez-Iglesias O, Ruiz-Llorente L, Sanchez-Martinez R, Garcia L, Zambrano A, et al (2008) Histone deacetylase inhibitors: mechanism of action and therapeutic use in cancer. Clin Transl Oncol 10: 395–398. 18628067 10.1007/s12094-008-0221-x

[8] Archin NM, Liberty AL, Kashuba AD, Choudhary SK, Kuruc JD, et al (2012) Administration of vorinostat disrupts HIV-1 latency in patients on antiretroviral therapy. Nature 487: 482–485. 22837004 10.1038/nature11286 PMC3704185

[9] Contreras X, Schweneker M, Chen CS, McCune JM, Deeks SG, et al (2009) Suberoylanilide hydroxamic acid reactivates HIV from latently infected cells. J Biol Chem 284: 6782–6789. 19136668 10.1074/jbc.M807898200 PMC2652322

[10] Ververis K, Karagiannis TC (2011) Potential non-oncological applications of histone deacetylase inhibitors. Am J Transl Res 3: 454–467. 22046487 PMC3204892

[11] Graff J, Kim D, Dobbin MM, Tsai LH (2011) Epigenetic regulation of gene expression in physiological and pathological brain processes. Physiol Rev 91: 603–649. 21527733 10.1152/physrev.00012.2010

[12] Graff J, Rei D, Guan JS, Wang WY, Seo J, et al (2012) An epigenetic blockade of cognitive functions in the neurodegenerating brain. Nature 483: 222–226. 22388814 10.1038/nature10849 PMC3498952

[13] Guan JS, Haggarty SJ, Giacometti E, Dannenberg JH, Joseph N, et al (2009) HDAC2 negatively regulates memory formation and synaptic plasticity. Nature 459: 55–60. 19424149 10.1038/nature07925 PMC3498958

[14] Rivieccio MA, Brochier C, Willis DE, Walker BA, D’Annibale MA, et al (2009) HDAC6 is a target for protection and regeneration following injury in the nervous system. Proc Natl Acad Sci U S A 106: 19599–19604. 19884510 10.1073/pnas.0907935106 PMC2780768

[15] Brunner D, Alexandrov V, Calderone B, Hanania T, Lowe D (2012) Behavior-Based Screening as an Approach to Polypharmacological Ligands. In: Peters J-U, editor. Polypharmacology in Drug Discovery. Hoboken, New Jersey: John Wiley & Sons, Inc. 301–309.

[16] Houghten RA, Pinilla C, Giulianotti MA, Appel JR, Dooley CT, et al (2008) Strategies for the use of mixture-based synthetic combinatorial libraries: scaffold ranking, direct testing in vivo, and enhanced deconvolution by computational methods. J Comb Chem 10: 3–19. 18067268 10.1021/cc7001205

[17] Roberds SL, Filippov I, Alexandrov V, Hanania T, Brunner D (2011) Rapid, computer vision-enabled murine screening system identifies neuropharmacological potential of two new mechanisms. Front Neurosci 5: 103. 21927596 10.3389/fnins.2011.00103 PMC3169783

[18] Hanson JE, Orr AL, Fernandez-Illescas S, Valenzuela RA, Madison DV (2010) Hippocampal Slice Cultures. Protocols for Neural Cell Culture: Humana Press. 299–311.

[19] Hanson JE, Deng L, Hackos DH, Lo SC, Lauffer BE, et al (2013) Histone Deacetylase 2 Cell Autonomously Suppresses Excitatory and Enhances Inhibitory Synaptic Function in CA1 Pyramidal Neurons. J Neurosci 33: 5924–5929. 23554474 10.1523/JNEUROSCI.3162-12.2013 PMC6618922

[20] Comery TA, Martone RL, Aschmies S, Atchison KP, Diamantidis G, et al (2005) Acute gamma-secretase inhibition improves contextual fear conditioning in the Tg2576 mouse model of Alzheimer’s disease. J Neurosci 25: 8898–8902. 16192379 10.1523/JNEUROSCI.2693-05.2005 PMC6725598

[21] Alarcon JM, Malleret G, Touzani K, Vronskaya S, Ishii S, et al (2004) Chromatin acetylation, memory, and LTP are impaired in CBP+/− mice: a model for the cognitive deficit in Rubinstein-Taybi syndrome and its amelioration. Neuron 42: 947–959. 15207239 10.1016/j.neuron.2004.05.021

[22] Levenson JM, O’Riordan KJ, Brown KD, Trinh MA, Molfese DL, et al (2004) Regulation of histone acetylation during memory formation in the hippocampus. J Biol Chem 279: 40545–40559. 15273246 10.1074/jbc.M402229200

[23] Vecsey CG, Hawk JD, Lattal KM, Stein JM, Fabian SA, et al (2007) Histone deacetylase inhibitors enhance memory and synaptic plasticity via CREB:CBP-dependent transcriptional activation. J Neurosci 27: 6128–6140. 17553985 10.1523/JNEUROSCI.0296-07.2007 PMC2925045

[24] Branco T, Staras K (2009) The probability of neurotransmitter release: variability and feedback control at single synapses. Nat Rev Neurosci 10: 373–383. 19377502 10.1038/nrn2634

[25] Bliss TV, Collingridge GL (1993) A synaptic model of memory: long-term potentiation in the hippocampus. Nature 361: 31–39. 8421494 10.1038/361031a0

[26] Martin SJ, Grimwood PD, Morris RG (2000) Synaptic plasticity and memory: an evaluation of the hypothesis. Annu Rev Neurosci 23: 649–711. 10845078 10.1146/annurev.neuro.23.1.649

[27] Kim JJ, Fanselow MS (1992) Modality-specific retrograde amnesia of fear. Science 256: 675–677. 1585183 10.1126/science.1585183

[28] Schenberg EE, Oliveira MG (2008) Effects of pre or posttraining dorsal hippocampus D-AP5 injection on fear conditioning to tone, background, and foreground context. Hippocampus 18: 1089–1093. 18727044 10.1002/hipo.20475

[29] Balducci C, Mehdawy B, Mare L, Giuliani A, Lorenzini L, et al (2011) The gamma-secretase modulator CHF5074 restores memory and hippocampal synaptic plasticity in plaque-free Tg2576 mice. J Alzheimers Dis 24: 799–816. 21321397 10.3233/JAD-2011-101839

[30] Kilgore M, Miller CA, Fass DM, Hennig KM, Haggarty SJ, et al (2010) Inhibitors of class 1 histone deacetylases reverse contextual memory deficits in a mouse model of Alzheimer’s disease. Neuropsychopharmacology 35: 870–880. 20010553 10.1038/npp.2009.197 PMC3055373

[31] Ricobaraza A, Cuadrado-Tejedor M, Marco S, Perez-Otano I, Garcia-Osta A (2012) Phenylbutyrate rescues dendritic spine loss associated with memory deficits in a mouse model of Alzheimer disease. Hippocampus 22: 1040–1050. 21069780 10.1002/hipo.20883

[32] Francis YI, Fa M, Ashraf H, Zhang H, Staniszewski A, et al (2009) Dysregulation of histone acetylation in the APP/PS1 mouse model of Alzheimer’s disease. J Alzheimers Dis 18: 131–139. 19625751 10.3233/JAD-2009-1134 PMC8962655

[33] Govindarajan N, Agis-Balboa RC, Walter J, Sananbenesi F, Fischer A (2011) Sodium butyrate improves memory function in an Alzheimer’s disease mouse model when administered at an advanced stage of disease progression. J Alzheimers Dis 26: 187–197. 21593570 10.3233/JAD-2011-110080

[34] Begley DJ (2004) ABC transporters and the blood-brain barrier. Curr Pharm Des 10: 1295–1312. 15134482 10.2174/1381612043384844

[35] Loscher W, Potschka H (2005) Blood-brain barrier active efflux transporters: ATP-binding cassette gene family. NeuroRx 2: 86–98. 15717060 10.1602/neurorx.2.1.86 PMC539326

[36] Hockly E, Richon VM, Woodman B, Smith DL, Zhou X, et al (2003) Suberoylanilide hydroxamic acid, a histone deacetylase inhibitor, ameliorates motor deficits in a mouse model of Huntington’s disease. Proc Natl Acad Sci U S A 100: 2041–2046. 12576549 10.1073/pnas.0437870100 PMC149955

[37] Bradner JE, West N, Grachan ML, Greenberg EF, Haggarty SJ, et al (2010) Chemical phylogenetics of histone deacetylases. Nat Chem Biol 6: 238–243. 20139990 10.1038/nchembio.313 PMC2822059

[38] Butler KV, Kozikowski AP (2008) Chemical origins of isoform selectivity in histone deacetylase inhibitors. Curr Pharm Des 14: 505–528. 18336297 10.2174/138161208783885353

[39] Tessier P, Smil DV, Wahhab A, Leit S, Rahil J, et al (2009) Diphenylmethylene hydroxamic acids as selective class IIa histone deacetylase inhibitors. Bioorg Med Chem Lett 19: 5684–5688. 19699639 10.1016/j.bmcl.2009.08.010

[40] Beconi M, Aziz O, Matthews K, Moumne L, O’Connell C, et al (2012) Oral administration of the pimelic diphenylamide HDAC inhibitor HDACi 4b is unsuitable for chronic inhibition of HDAC activity in the CNS in vivo. PLoS One 7: e44498. 22973455 10.1371/journal.pone.0044498 PMC3433414

[41] Fass DM, Reis SA, Ghosh B, Hennig KM, Joseph NF, et al.. (2013) Crebinostat: A novel cognitive enhancer that inhibits histone deacetylase activity and modulates chromatin-mediated neuroplasticity. Neuropharmacology. 10.1016/j.neuropharm.2012.06.043 PMC3447535 22771460

[42] Fischer A, Sananbenesi F, Wang X, Dobbin M, Tsai LH (2007) Recovery of learning and memory is associated with chromatin remodelling. Nature 447: 178–182. 17468743 10.1038/nature05772

[43] Peleg S, Sananbenesi F, Zovoilis A, Burkhardt S, Bahari-Javan S, et al (2010) Altered histone acetylation is associated with age-dependent memory impairment in mice. Science 328: 753–756. 20448184 10.1126/science.1186088

[44] Spiller SE, Ditzler SH, Pullar BJ, Olson JM (2008) Response of preclinical medulloblastoma models to combination therapy with 13-cis retinoic acid and suberoylanilide hydroxamic acid (SAHA). J Neurooncol 87: 133–141. 18060600 10.1007/s11060-007-9505-1

[45] Yin D, Ong JM, Hu J, Desmond JC, Kawamata N, et al (2007) Suberoylanilide hydroxamic acid, a histone deacetylase inhibitor: effects on gene expression and growth of glioma cells in vitro and in vivo. Clin Cancer Res 13: 1045–1052. 17289901 10.1158/1078-0432.CCR-06-1261

[46] Lee J, Lund-Smith C, Borboa A, Gonzalez AM, Baird A, et al (2009) Glioma-induced remodeling of the neurovascular unit. Brain Res 1288: 125–134. 19595677 10.1016/j.brainres.2009.06.095 PMC2735571

[47] Lund CV, Nguyen MT, Owens GC, Pakchoian AJ, Shaterian A, et al (2006) Reduced glioma infiltration in Src-deficient mice. J Neurooncol 78: 19–29. 16552622 10.1007/s11060-005-9068-y PMC4002283

[48] Liner KJ, 2nd, Ro MJ, Robertson KR (2010) HIV, antiretroviral therapies, and the brain. Curr HIV/AIDS Rep 7: 85–91. 20425562 10.1007/s11904-010-0042-8

[49] Thompson KA, Cherry CL, Bell JE, McLean CA (2011) Brain cell reservoirs of latent virus in presymptomatic HIV-infected individuals. Am J Pathol 179: 1623–1629. 21871429 10.1016/j.ajpath.2011.06.039 PMC3181362

